# Social cohesion in contexts of violence against women: evidence from Honduras and implications for public policy

**DOI:** 10.3389/fsoc.2026.1925726

**Published:** 2026-10-07

**Authors:** Josué Sabillón-Casco, Darlis Banegas-Maldonado, Héctor Moncada-Múnoz

**Affiliations:** 1 Department of Sociology, National Autonomous University of Honduras (UNAH), Tegucigalpa, Honduras; 2 Doctoral Program in Social Sciences on Central America, Universidad de Costa Rica (UCR), San José, Costa Rica

**Keywords:** Central America, community participation, public administration, social policy, women

## Abstract

This cross-sectional, descriptive study assesses social cohesion among women in Honduras using microdata from the 2022 First National Specialized Survey on Violence Against Women and Girls (ENESVMN). Utilizing a multidimensional framework, the analysis examines relational, institutional, and normative variables based on an effective sample of 5,962 interviewed women. The normalized-weight results show that 27.1% (nominal 95% CI: 26.02–28.28) of participants exhibit high social cohesion, 26.6% (95% CI: 25.50–27.74) exhibit moderate cohesion, and 46.3% (95% CI: 45.00–47.53) exhibit low cohesion. On a municipal scale, the Central District concentrates the highest proportions across all cohesion levels, at 19.1%, 14.7%, and 12.4%, respectively. These findings show cohesion divided fairly evenly between high and moderate levels, with low cohesion as the single largest category within the analyzed sample, a pattern that the literature on welfare regimes in Central America associates with precarious social stability under conditions of structural violence and institutional absence. Building on this evidence and the broader literature on welfare regimes in Central America, the study argues that strengthening social cohesion is likely to require a more redistributive public sector, including fiscal reform. Such structural change, supported by a progressive tax system, is essential to facilitate effective wealth redistribution through social policies that have remained historically underdeveloped in the country. These results contribute to the literature on social order in high-violence contexts and offer critical insights for demographic policy and public management in Central America.

## Introduction

The 2022 First National Specialized Survey on Violence against Women and Girls (ENESVMN) in Honduras estimated that 52.8% of respondents have experienced at least one incident of violence during their lifetime. Regarding violence suffered within the 12 months prior to the survey, 23.2% reported being victims of at least one violent act. Among the most common forms, psychological violence is the most prevalent (39.5%), followed by patrimonial, economic, or discriminatory violence (26.4%), sexual violence (23.7%), and physical violence (18.2%) [National Institute of Statistics (INE), 2022].

Within this context, what is the estimated level of social cohesion among women in Honduras? To address this question, this study employs the social cohesion construct developed by Mora Salas (2015), which is cross-referenced with the ENESVMN questionnaire to identify suitable proxy variables. Once defined, the microdata are dichotomized to construct an aggregate estimation of the level of social cohesion. Additionally, estimates are presented for 10 of the country's 298 municipalities. These results aim to contribute to both the literature on social cohesion in violent contexts and to the field of public administration, particularly regarding the role of the state at the national and municipal levels.

Crucially, the analysis of social cohesion is inextricably linked to the role of the state. The state performs public functions that directly impact individual and family life, notably in three key areas: the regulation of behavior through norms, laws, and public policies; the collection of resources through taxation; and the provision of public goods and services such as education, healthcare, and social security (Kamerman and Kahn, 1978). These functions establish the structural conditions under which social bonds are either forged or eroded.

Furthermore, existing literature has demonstrated that in Honduras, violence—particularly against women and young men from impoverished sectors—cannot be eradicated solely through legal or institutional reforms. Persistent structural, cultural, and symbolic factors continue to reproduce direct violence (Sabillón Casco, 2025b), suggesting that state capacity to strengthen social cohesion through formal interventions alone is inherently limited.

This situation is exacerbated by the social organization of welfare. In Honduras, women disproportionately shoulder the responsibility for sustaining wellbeing within a context characterized by state weakness, precarious labor markets (Sabillón Casco et al., 2018), and limited male co-responsibility in caregiving (Martínez Franzoni, 2021). Added to this is the impact of punitive security policies in Central America, which, by facilitating the mass incarceration of men, reinforce the overburdening of women who frequently serve as heads of household (Martínez Franzoni et al., 2025).

From this perspective, social cohesion should be understood not only as a social outcome but also as an indicator of the effectiveness and limitations of public service. In contexts of pervasive violence and highly familialist welfare regimes (Martínez Franzoni and Sánchez-Ancochea, 2021), such as the Honduran case, its estimation allows for an evaluation of the extent to which the state successfully articulates social ties, redistributes risks, and maintains minimum conditions for social integration through dimensions such as social bonds, trust, sense of belonging, and shared values (Mora Salas, 2015).

Throughout the analysis, violence against women is treated as the structural context within which social cohesion is estimated, not as a directly tested explanatory factor. The descriptive, cross-sectional design of this study does not support causal or fully controlled associational claims linking individual violence exposure to the cohesion score, and the ENESVMN was not designed to allow an item-level test of that relationship. Where the Discussion and Conclusion draw on violence to interpret the observed distribution of cohesion, this reflects a theoretically grounded reading consistent with the cited literature, not a direct empirical finding of the present analysis.

The article is organized into four primary sections following this introduction. The first section establishes the ‘Background', encompassing the theoretical framework predicated on the Mora Salas (2015) approach, the state of the art, and the specific Honduran context. The second details the “Data and Methods” utilized in the study. The third presents the Section Results, covering both the general national estimation and the municipal-level analysis. Finally, the fourth section provides the Section Discussion and conclusion, addressing the primary findings, their implications for public policy, and the study's final synthesis.

## Background

### Theoretical framework

Mora Salas (2015) defines social cohesion as the

“Existence of a structure of social ties, the disposition of individuals to maintain and renew said bonds, identification with the collectivity, and the presence of shared values” (p. 117)

This definition allows social cohesion to be conceived as an attribute of collectivities, rather than individuals; it is observable in varying degrees and is analytically distinguishable from both its causes and its consequences.

From this perspective, social cohesion constitutes a multidimensional phenomenon that articulates both objective and subjective dimensions of social life. On one hand, it refers to the existence of social bonds produced and reproduced within primary, secondary, and tertiary spheres; on the other, it incorporates subjective elements such as trust, shared values (solidarity, cooperation, altruism, and tolerance), and a sense of belonging to a collectivity. This formulation overcomes normative approaches that tend to equate cohesion with a “good society,” highlighting that cohesive configurations can exist even under exclusionary institutional arrangements or value systems (Mora Salas, 2015, p. 125).

Based on this conceptual delimitation, it is possible to reconstruct the classical sociological problem underlying the concept of social cohesion: the inquiry into the foundations of social order. Within this foundational debate, liberal perspectives argued that society is held together through instrumental agreements among rational individuals, whereas contractualist schools emphasized the existence of shared norms and values as a prerequisite for social life. Within this framework, Auguste Comte (1798–1857) questioned the sufficiency of the market as an integrative mechanism, underscoring the role of a moral system of beliefs, while Herbert Spencer (1820–1903) defended the idea of spontaneous integration based on the equilibrium of individual contracts.

The most systematic development of the concept is found in the work of Émile Durkheim (1858–1917), who established the analytical foundations of social cohesion by linking it to the nature of solidarity within different types of society. In *The Division of Labor in Society* and *Suicide*, Durkheim posits that contractual relations cannot be sustained on their own; rather, they require a prior moral basis to regulate social interaction (Mora Salas, 2015, p. 16).

In this sense, Durkheim distinguishes between two forms of solidarity. Mechanical solidarity, characteristic of traditional societies with low social differentiation, is predicated on a strong collective conscience and the similarity between individuals. In contrast, organic solidarity—proper to modern societies—emerges from the social division of labor and is based on the functional interdependence between differentiated individuals. Throughout this transition, cohesion does not vanish but is instead transformed; it shifts from being rooted in homogeneity to depending on social institutions that regulate cooperation and disseminate shared values.

Subsequently, the concept was reformulated within the framework of structural-functionalism, particularly in the work of Talcott Parsons (1902–1979), who integrated it under the notion of “social integration” as part of the problem of order (p. 19). However, for several decades, social cohesion lost its analytical centrality until its resurgence in the 1990s, driven by international organizations such as the European Union, the Organization for Economic Co-operation and Development (OECD), and the Economic Commission for Latin America and the Caribbean (ECLAC/CEPAL). This return was marked by a frequently normative and pragmatic usage of the concept, in which cohesion tended to be conflated with objectives such as equity or economic growth (Mora Salas, 2015, p. 21–23).

To address this conceptual ambiguity, an analytical delimitation is introduced that clearly distinguishes between social cohesion and its conditioning factors. Specifically, this perspective maintains that inequality does not constitute a defining component of cohesion but is rather a factor that can influence it by either eroding or strengthening it (p. 127)—a relationship that must be established empirically. Furthermore, it posits that social cohesion is a gradational phenomenon (p. 133), implying that societies may exhibit varying levels of cohesion based on their historical and structural configurations.

This conceptual delimitation highlights a critical distinction: the presence of personal ties, mutual trust, and shared values represents a resource potential, but does not by itself guarantee a community's capacity to intervene in shared problems. Following Sampson et al. (1997) and Morenoff et al. (2001), collective efficacy designates precisely this activated capacity, defined as the linkage of mutual trust with a shared willingness among residents to exercise informal social control for the common good. Social cohesion and collective efficacy are therefore distinct, non-equivalent constructs: cohesion describes the presence of network ties and solidarity, whereas collective efficacy captures the agentic dimension through which those resources translate into demonstrated collective action. This distinction is central to the operationalization presented below, as the ENESVMN proxies approximate the structural presence of social cohesion rather than the active exercise of informal control.

Because the operationalization draws on items referring to social security coverage, police and court help-seeking, and community infrastructure, it is also necessary to situate the relationship between communities and institutions within this framework. Institutional trust, geographic and administrative proximity to state services, and the perceived capacity of institutions to act are conceptually distinct from interpersonal cohesion, even though both may covary in contexts of state weakness. In what follows, items capturing institutional access or institutional help-seeking are treated analytically as proxies for institutional trust and institutional presence, and are interpreted separately from proxies capturing interpersonal ties, trust, belonging, and shared values.

Alternatively, some authors position the individual as the central agent of change, highlighting participation as the articulating axis of social life. In this regard, they revalue work as a source of integration and human fulfillment, while the territorial community is proposed as a key space for rebuilding social bonds in the face of contemporary fragmentation. Similarly, a “facilitator state” role is proposed, oriented toward participatory governance, conceiving cohesion as a tool of resistance against neoliberal logics (Martínez Betancourt and Neira Milian, 2021).

This theoretical framework serves as a robust analytical tool for examining contexts of violence against women, as it facilitates an exploration of how such violence affects both the structural configuration of social ties and the subjective dimensions underpinning collective life. Specifically, pervasive violence can erode interpersonal and institutional trust, weaken values of cooperation and solidarity, and fragment the sense of belonging, thereby fostering environments characterized by low social cohesion. Concurrently, the gradational approach proposed by Mora Salas (2015) provides a pathway for identifying moderate levels of cohesion which, despite their inherent instability, represent critical opportunities for public intervention aimed at reinforcing social bonds and narrowing gaps of vulnerability.

### State of the art

In recent literature, social cohesion has evolved from a broad and diffuse notion of social order into a more precise, dynamic, and methodologically sophisticated research field. Within this context, a primary innovation involves its conceptualization as a multilevel phenomenon (micro, meso, and macro), which allows for the articulation of individual experiences, group differences, and aggregate societal properties. This approach identifies social cohesion not only as a social outcome but also as a factor influencing social processes, while enabling the development of measurement strategies capable of capturing both internal differences and cross-societal comparisons (Aruqaj, 2023).

Alongside this measurement focus, another significant innovation is the movement toward operationalizing social cohesion through scales, indices, and statistical models applied to specific contexts. For example, a Social Cohesion Index was developed in marginalized areas of Mexico to evaluate the impact of social programs, demonstrating that cohesion can serve as a public policy evaluation tool. Researchers found that targeted interventions do not necessarily yield significant changes in social cohesion, reinforcing the idea that it is a structurally grounded phenomenon (Mosqueda Tapia, 2023). Similarly, Candia Cid et al. (2022) shifted the focus to the territorial level through a psychometric scale applied at the municipal level in Chile, validating dimensions such as trust, belonging, participation, and support networks. This line of inquiry demonstrates that cohesion is no longer studied solely on a national scale but also within subnational spaces, where local inequalities, crises of coexistence, and opportunities for public intervention are most pronounced.

A further novel line in contemporary debate lies in the reconceptualization of social cohesion as a dynamic and historically situated process. This perspective moves away from static definitions, proposing instead that cohesion be understood as a socio-historical construction in permanent recomposition—influenced by concrete structural conditions and sustained by the action and participation of individuals. This framework is vital because it incorporates conflict not as an anomaly external to cohesion, but as one of its constitutive elements. From this viewpoint, there is no universal model of cohesion; rather, diverse configurations exist that depend on historical trajectories, inequalities, institutional arrangements, and specific forms of social interaction (Martínez Betancourt and Neira Milian, 2021).

Furthermore, recent scholarship suggests treating social cohesion as an emergent and non-linear phenomenon. Utilizing agent-based models calibrated with empirical evidence, researchers have demonstrated that cohesion and trust arise from social interaction, are bolstered through cooperation, and are undermined by conflict. Their proposal emphasizes that cohesion should be understood not merely as a fixed structure but as a relational and adaptive dynamic, particularly sensitive to group size, interaction frequency, and the capacity for reconstruction following conflictive episodes. This finding is significant as it frames cohesion not solely as a “social stock” but as a process susceptible to erosion or recomposition depending on evolving conditions of coexistence (Feinberg et al., 2023).

Similarly, the work of Briceño-León and Camardiel (2021) demonstrates that violence erodes cohesion not only through its material effects but primarily through the fear it generates and the subsequent loss of trust in security and justice institutions. Their proposal to operationalize cohesion through coexistence, normativity, and wellbeing provides a framework particularly suited for research in Latin American contexts characterized by insecurity and institutional decay. Moreover, their finding that daily crimes have a greater impact on the perception of cohesion than major crimes is highly suggestive; it implies that the erosion of social ties depends not only on extreme events but on the daily repetition of threats, fears, and lack of protection (Briceño-León and Camardiel, 2021).

In a similar vein, Quiroga Juárez and Villafuerte Valdés (2023) introduce a novel contribution by integrating social cohesion, development, and wellbeing from the perspective of citizen perception. Their model, constructed using AmericasBarometer data, shows that cohesion can be structured around dimensions such as governmental legitimacy and effectiveness, state performance, and perceptions of authoritarianism. This approach broadens the analytical field by situating cohesion at the interface of society, the state, and public opinion. Consequently, cohesion transcends simple interpersonal ties and emerges as an expression of institutional trust, governmental evaluation, and normative expectations regarding the political order (Quiroga Juárez and Villafuerte Valdés, 2023).

A closely related line of research, more directly concerned with the mechanisms linking community relations to violence, is the social-disorganization and collective-efficacy tradition. Sampson et al. (1997) show that neighborhood-level collective efficacy, rather than social ties alone, predicts lower rates of violent crime, while Morenoff et al. (2001) extend this finding to demonstrate how neighborhood inequality and spatial dynamics condition collective efficacy's protective effect. Applications to Latin American contexts, such as Carneiro et al. (2023) on the urban space and territorial distribution of violent crime in Brazilian municipalities, situate this theoretical framework within the region's high-violence urban settings, providing a useful comparative anchor for the territorial analysis presented below.

The most recent literature also advances the clarification of the temporality and causality of cohesion. Evidence suggests a bidirectional and causal relationship between social cohesion and volunteering, particularly within the horizontal dimension of cohesion. This result is crucial for distinguishing cohesion from its effects and antecedents, showing that it can be reinforced through concrete participatory practices, even if different dimensions behave disparately. The distinction between horizontal and vertical cohesion is likewise useful, as it allows for the separate consideration of interpersonal ties and trust in institutions—two planes that may deteriorate differently in contexts of violence against women (Davies et al., 2024).

Finally, a systematic PRISMA review confirmed that social cohesion has consolidated as a multidimensional concept whose recent study converges around variables such as trust, participation, sense of belonging, and social ties. This review also identifies a prevailing trend: the field is moving away from purely conceptual discussions toward the construction of indicators, scales, and comparative models, while increasingly linking cohesion to inequality, poverty, political crises, and public policy (Lozano Fernández, 2025).

### The Honduran context for women

Women in Honduras are embedded within what is defined as an informal, highly familialist welfare regime (Martínez Franzoni, 2005; Martínez Franzoni and Sánchez-Ancochea, 2021). This model is characterized by marginal state provision of social protection and a massive transfer of risks to the private sphere. Within this framework, the Honduran state positions itself as a laggard in indicators of coverage, generosity, and equity for fundamental services such as healthcare and pensions (Martínez Franzoni, 2008). Faced with this institutional weakness, women assume a dual role: as providers of wellbeing and as the primary articulators of the scarce resources obtained from the market and state spheres.

This pursuit of wellbeing takes place within a labor market marked by structural heterogeneity and precariousness, where belonging to the formal sector does not guarantee dignified conditions, as it also presents high levels of instability and low wages (Sabillón Casco et al., 2018). For the majority, labor informality is not merely a lack of a contract, but a survival and entrepreneurial strategy against markets that are incapable of absorbing the labor force (Sabillón Casco, 2024a). In this context, female household headship emerges as both a demographic shift and a response to the absence of male, state, and market co-responsibility (Martínez Franzoni, 2021).

When licit mechanisms fail, extreme strategies such as “clandestine welfare” emerge (Zapata Celestino, 2023). In communities where the state maintains a “policy of absence,” illicit actors (such as gangs and maras) create parallel protection networks, intervening directly in the provision of goods or enforcing price controls and curfews under a logic of criminal governance. Furthermore, the care economy is sustained externally through remittances which, far from being a structural solution, reinforce the familialization and feminization of care, as they are mostly sent to women to cover basic health and education needs that the Honduran state chooses to ignore (Sabillón Casco, 2024b).

Finally, this environment is aggravated by “gender necropolitics,” where political and social power regulates the lives and deaths of women, treating them as disposable bodies through femicides and impunity (Sagot Rodríguez, 2024; p. 15). Honduras presents a convergence of four forms of violence (direct, structural, cultural, and symbolic) (Sabillón Casco, 2025b) that fracture social cohesion and compel processes of systematic expulsion (Sabillón Casco, 2025a), forcing women to seek abroad the wellbeing that their own country denies them.

The concepts introduced in this section, policy of absence, clandestine welfare, and gender necropolitics, are retained here as background literature describing the structural and institutional context in which the ENESVMN was fielded. They are not treated as directly measured or statistically tested explanatory mechanisms of the cohesion levels reported in the Results; where the Discussion returns to them, it does so as theoretically grounded interpretation rather than as a claim tested by the present descriptive analysis.

### Data and methods

This descriptive, cross-sectional study utilizes microdata from the 2022 First National Specialized Survey on Violence Against Women and Girls (ENESVMN)^1^ in Honduras. Given that the database was not originally designed to estimate social cohesion, a multidimensional estimation was constructed based on available variables that capture the relational, institutional, and normative dimensions associated with the theoretical framework proposed by Mora Salas (2015). A total of 14 proxy variables were dichotomized into “cohesion” and “no cohesion” categories; the methodological decisions adopted for these classifications are presented below:

The decision to utilize these microdata as a secondary source for estimating social cohesion should be understood as a pragmatic response to the “empirical poverty” prevalent in the region (Mora Salas, 2015, p. 156), particularly given the absence of a database specifically designed to measure this concept. Accordingly, the methodological decisions adopted aim to capture, to the greatest extent possible, key dimensions such as social bonds, trust, sense of belonging, and shared values.

The index is built from the fourteen items in Table 1, selected because each maps onto one of the four dimensions in the conceptual framework proposed by Mora Salas (2015) for measuring social cohesion under conditions of empirical poverty, vínculos sociales (social bonds), confianza (trust), valores compartidos (shared values), and sentido de pertenencia (sense of belonging). Following the same framework, the items are drawn from its three variable types, behavior (frequency of contact with family, friends, neighbors, or affinity groups), opinion (the expectation or valuation of support received from them, and trust in institutions), and attitude (solidarity, cooperation, altruism, and tolerance). The fourteen items are each dichotomized into a “no-cohesion” flag (1) or a “cohesion” flag (0) following the coding shown in Table 1, using the primary coding of the two partner-referencing participation items (MC1107_5, MC1107_7); the flags are summed with equal item weights, into a single score ranging from 4 to 13 in the analyzed sample. Missing or routed-out responses are handled item-by-item rather than uniformly.

**Table 1:** Social cohesion dimensions proposed by Mora Salas (2015) and proxy survey items for Honduras (actual sample *N* = 5,962).

Dimension	Component	Variable	Associated survey items	Categories and dichotomization	% (*n*^a^)
*Social ties*: existence of social bonds formed through the individual's daily interaction with other people.	*Existence of social bonds*: extent of the individual's social network based on spaces of high social interaction.	*Family ties*: investigates the frequency of relationships with the closest family members.	HL3. Relationship to head of household^b^	*Cohesion = 0:* 1. Head of household *No cohesion = 1:* 2. Spouse or partner, 3. Son/daughter, 4. Stepchild, 5. Father/Mother, 6. Brother/Sister, 7. Son/Daughter-in-law, 8. Other relative (grandchild, grandparent, uncle/aunt, cousin, etc.), 9. Other non-relative (father/mother-in-law, brother/sister-in-law, guest, etc.), 10. Domestic worker	32.7 (1,948)
		*Neighborhood ties:* investigates the frequency of relationships within a circle characterized by shared socio-territorial proximity.	ACOM553. When there are problems in your community, do you talk, converse, or ask for help?	*Cohesion = 0* 1. Si *No cohesion = 1* 2. No 3. *Other = 1*	6.8 (407)
			ACOM554_1. With whom do you talk, converse, or ask for help?	*Cohesion = 0* 1. Relatives, 2. Friends, 3. Neighbors *No cohesion = 1* 7. Priest, pastor, or religious leader, 9. Health facility (doctor, health personnel), 10. National Police, 11. Human resources, supervisor, direct boss, or colleague, 12. Other (specify) *Other = 1*	6.4 (384)
*Trust:* the expectation that others (whether close or distant) will conduct themselves in their dealings with the individual according to a set of values and obligations, resulting in non-conflictive interaction.	*Specific trust:* a qualitative assessment of previously established social ties in terms of reciprocity and expectations.	*Expectation of support from close friends:* assesses the degree to which close friends are trusted to provide support if the individual requires it.	ACOM549. Even though you mentioned you did not seek help, did anyone provide help to you?	*Cohesion = 0* 1. Yes *No cohesion = 1* 2. No *Other = 1*	3.0 (178)
	*Institutional trust:* the individual's assessment of public institutions, perceiving them as part of an institutional system or network through which the community—and specifically the state—assumes responsibilities and commitments to its members.	*Sense of protection in the event of illness:* explores the individual's sense of protection provided by the public health service.	AE207_1. Is (name) affiliated with or covered by any of the following public or private social security systems? (option 1)	*Cohesion = 0* 1. RAP, 2. INJUPEMP, 3. INPREMA, 4. IPM, 5. IHSS Member, 6. IHSS Beneficiary, 7. Private pension fund, 8. Private medical insurance, 9. Union, 10. Guild or workers' association, 12. Other (specify) *No cohesion = 1* 11. None of the above *Other = 1*	11.7 (696)
		*Sense of protection in the event of insecurity:* explores the individual's sense of protection provided by citizen security institutions.	BAYUD_VIO_PN. In response to situations of violence, did you seek help from the National Police?	*Cohesion = 0* 1. Si *No cohesion = 1* 0. No *Other = 1*	2.7 (162)
			BAYUD_VIO_JZ. In response to situations of violence, did you seek help from the Court?	*Cohesion = 0* 1. Si *No cohesion = 1* 0. No *Other = 1*	1.3 (77)
			MC1102. Do you know of laws that protect women's rights?	*Cohesion = 0* 1. Si *No cohesion = 1* 2. No *Other = 1*	28.0 (1,667)
*Sense of belonging*: the perception of being part of and feeling integrated into a social collectivity, as experienced by the members of a society.	*Identification:* refers to the collectivities or aggregates to which the individual feels they belong.	*Individual sense of identification with the family:* captures the individual's identification with their family unit.	MC1107_5. If you want to participate in a neighborhood or political activity, [what is your protocol regarding] your husband or partner?^c^	*Cohesion = 0* 2. Informs him or asks for his opinion, 3. Does not go alone; goes with him, 4. Does not need to do anything/Does not do it, 5. Other (specify), 6. Not applicable *No cohesion = 1* 1. Must ask for permission *Missing or other unlisted response = 1*	37.6 (2,241)
	**Sense of identification with the community:** Captures the individual's identification with their local community.		ACOM555_1. Due to the situations experienced, did you encounter: (Option 1)	*Cohesion* **= 0** 9. Did not experience any problems *No cohesion = 1* 1. Ceasing to transit through that area, 2. Disinterest in community activities, 3. Relocating or changing residence, 4. Family problems, 5. Depression or anxiety, 6. Distress or fear, 7.Headaches, 8. Sleep difficulties or disturbances (insomnia or excessive sleep) *Other = 0*	90.8 (5,412)
	DV306A. Does your community have public street lighting services?			*Cohesion = 0* 1. Si *No cohesion = 1* 2. No *Other = 1*	85.6 (5,105)
*Shared values:* a set of general motivations orienting the attitudes, opinions, and behaviors or actions of individuals.	*Tolerance as a value:* a value that orients individual action toward a disposition for establishing ties and interactions.	*Socioeconomic discrimination:* captures, through individual perception of being discriminated against for socioeconomic reasons, the existence or incidence of tolerance as a value in others.	DISCR_LAB. Percentage of women aged 15 and older who have experienced labor discrimination during their lifetime.	*Cohesion = 0* 0. Has not experienced labor discrimination *No cohesion = 1* 1. Has experienced labor discrimination *Other = 1*	46.4 (2,769)
		VTC1204. At home, who has absorbed the majority of the additional domestic workload?		*Cohesion = 0* 7. It is distributed equitably *No cohesion = 1* 1. Yourself, 2. Your husband or partner, 3. Your daughter/son, 4. Mother, 5. Father, 6. Other family member *Other = 1*	30.8 (1,838)
		*Political discrimination:* captures, through individual perception of being discriminated against due to political preference, the existence or incidence of tolerance as a value in others.	MC1107_7. To vote for a political party or candidate, [what is your protocol regarding] your husband or partner?	*Cohesion = 0* 3. Does not go alone; goes with him, 4. Does not need to do anything/Does not do it, 6. Not applicable *No cohesion = 1* 1. Must ask for permission, 2. Informs him or asks for his opinion, 5. Other (specify) *Other = 0*	85.2 (5,081)

**Source:** Own elaboration based on the proposal by Mora Salas (2015) and items from the 2022 ENESVMN questionnaire.

Percentages and *n* values refer to the cohesion-coded category (0). Counts are normalized weighted counts, rounded for display, rather than observed respondent counts. The coding of missing and routed-out responses follows the item-specific rules described below.

^a^Only the percentage and sample size of the socially cohesive group are reported for each variable.

^*b*^Female-headed households are often considered indicators of women's empowerment. While these women may face a lack of co-responsibility from their husbands or partners, a study conducted in Honduras among domestic workers found that female heads of household earned even higher incomes than married women (Sabillón Casco, 2024a, 134, 135).

^c^Within the context of intimate partnerships, social cohesion is assumed to be reflected in communication and shared decision-making processes, including consulting a partner or taking their views into account, as well as participating in activities together. Such practices are understood as expressions of social bonds, coordination, and interpersonal closeness in everyday life.

Missing or routed-out responses were assigned a score of 1 in twelve items and a score of 0 in ACOM555_1 and MC1107_7. For MC1107_5, codes 2–6 were scored 0, while code 1 and missing responses were scored 1. Consequently, all respondents received fourteen computed item flags. These are assigned scores rather than fourteen observed substantive responses. A separate indicator distinguishes substantive responses to MC1107_5 (codes 1–5; 2,511 observed respondents) from not-applicable or missing responses (3,451 observed respondents); it does not exclude an item from the primary score. The normalized-weight shares of these subgroups are 40.5% and 59.5%, respectively. Their implications for score comparability are examined in Section Limitations below.

Respondents are classified as high cohesion (score 4–8), moderate cohesion (score 9), or low cohesion (score 10 or higher). These are fixed analytic cut-points rather than an externally validated typology. The observed fourteen-item range is 4–13, with midpoint 8.5; the upper of the two nearest integers, 9, was selected as a single-score moderate category. The historical sixteen-item specification used a different classification scheme, with three four-value bands over its observed range of 4–15 (high 4–7, moderate 8–11, low 12–15). Comparisons between these specifications therefore reflect both item inclusion and the different classification thresholds. Tables 2, 3 use the fourteen-item primary classification; the alternative partner-item coding and the sixteen-item specification are reported in the Section Sensitivity analysis below.

Table 2General and disaggregated estimation of social cohesion.

**Table:** 

**General estimation of cohesion**									
Level	High			Moderate			Low		
	*n*	%	(95% CI)	*n*	%	(95% CI)	*n*	%	(95% CI)
	1,618	27.1	(26.02–28.28)	1,586	26.6	(25.50–27.74)	2,758	46.3	(45.00–47.53)

**Table:** 

Disaggregated estimation						
Level	*n*	%	*n*	%	*n*	%
**Educational levelS** ^a^						
Primary	615	40.7	828	58.3	1,396	59.1
Secondary	581	38.5	481	33.8	802	33.9
Higher	315	20.8	112	7.9	165	7.0
**Marital status**						
Married/cohabiting	1,003	62.0	871	54.9	1,524	55.3
Other marital status	477	29.5	528	33.3	1,069	38.8
Widowed	138	8.5	188	11.8	165	6.0
**Age groups**						
15 to 29	375	23.2	408	25.7	972	35.2
30 to 64	1,020	63.0	927	58.4	1,434	52.0
65 and older	223	13.8	251	15.8	352	12.8

**Source:** Own elaboration based on ENESVMN microdata.

*n* denotes normalized weighted counts, rounded for display; percentages in the disaggregated panel describe the composition of each cohesion level. In the subgroup cross-tabulations, weighted cells were rounded before calculating percentages, so their totals may differ from separately rounded general counts. The actual sample contains 5,962 respondents. The 95% confidence intervals, reported only for the general estimates, are nominal Wilson intervals and do not adjust for the complex sampling design. Education includes 0–6, 7–12, and 13–20 years of schooling; other values are excluded from that subgroup table. Other marital status includes divorced, separated, single and unknown responses (EstCivil codes 3, 4, 6, and 9).

**Note on interpretation:** the percentages in the disaggregated panel above report the composition of each cohesion level by education, marital status, and age (i.e., what share of, say, the high-cohesion group is married), not the within-group prevalence of cohesion levels (i.e., what share of married women fall into each level). Within-group prevalence estimates, computed using the classification procedure described in the Section Data and Methods above, are as follows. All subgroup *n* values below are sums of rounded normalized weighted cells, not observed numbers of respondents. By educational level: among women with primary schooling (*n* = 2,839), 21.7% score high, 29.2% moderate, and 49.2% low cohesion; among those with secondary schooling (*n* = 1,864), 31.2% high, 25.8% moderate, 43.0% low; among those with higher education (*n* = 592), 53.2% high, 18.9% moderate, 27.9% low. By marital status: among married or cohabiting women (*n* = 3,398), 29.5% score high, 25.6% moderate, 44.8% low; among respondents in the other marital-status group (*n* = 2,074), 23.0% high, 25.5% moderate, 51.5% low; among widowed women (*n* = 491), 28.1% high, 38.3% moderate, 33.6% low. Because two of the fourteen index items reference a partner, these marital-status figures cannot be fully separated from the index's own construction, and are reported here descriptively rather than as an independent finding about marital status (see Section Discussion and Limitations). By age group: among women aged 15–29 (*n* = 1,755), 21.4% score high, 23.2% moderate, and 55.4% low cohesion; among women aged 30–64 (*n* = 3,381), 30.2% high, 27.4% moderate, 42.4% low; among women aged 65 and older (*n* = 826), 27.0% high, 30.4% moderate, 42.6% low. These prevalence figures are consistent with the pattern already visible in the composition percentages above (higher cohesion concentrated among more educated, and 30–64-year-old women), while additionally showing, within each group, that a substantial minority-to-majority share falls in the low-cohesion category, most markedly among women aged 15–29 (55.4%) and the other marital-status group (51.5%). No inferential tests (e.g., chi-square, logistic regression) have been run on these differences; they are reported descriptively, as proxy-index patterns rather than as independently validated levels of social cohesion. A formal significance test is identified as a next step for future work.

^a^For each of the three educational levels (primary, secondary, and tertiary), women were classified according to the highest level attained, regardless of whether it was completed. Accordingly, both complete and incomplete educational trajectories are included. For instance, a woman with 7 years of schooling is categorized at the secondary level, even if she attended only the first year of secondary education.

**Table 3:** Social cohesion estimation by municipality.

Municipality^a^	High		Moderate		Low	
	*n*	%	*n*	%	*n*	%
Central district	310	19.1	234	14.7	343	12.4
San Pedro Sula	185	11.4	162	10.2	201	7.3
La Ceiba	58	3.6	46	2.9	42	1.5
Comayagua	23	1.4	32	2.0	30	1.1
Choloma	78	4.8	37	2.3	73	2.6
Danlí	13	0.8	33	2.1	65	2.4
Gracias	7	0.4	4	0.3	16	0.6
Juticalpa	18	1.1	32	2.0	54	2.0
El Progreso	47	2.9	62	3.9	61	2.2
Olanchito	26	1.6	28	1.8	50	1.8
Remaining municipalities	854	52.7	918	57.8	1,825	66.1

**Source:** Own elaboration based on 2022 ENESVMN microdata.

*n* denotes normalized weighted counts, rounded for display, rather than observed respondent counts. Percentages describe the composition of each cohesion level. Weighted cell counts were rounded before calculating percentages; their sums can therefore differ from the general counts in Table 2.

**Note on interpretation:** as with the disaggregated panel above, the percentages reported by municipality describe the composition of each cohesion level (the share of, e.g., the low-cohesion group located in a given municipality), not the within-municipality prevalence of cohesion levels. Given the survey's prioritized-municipality sampling design (discussed below), these figures should not be read as evidence of comparative territorial risk.

^a^Detailed municipal sociodemographic profiles for Honduras are available at: https://oee.unah.edu.hn/lineas-de-investigaci/desarrollo-economico-con-enfoque-territorial/psmh.

A sensitivity analysis of the recoding of the two partner-referencing participation items (MC1107_5, MC1107_7) is reported in the Section Results below. Furthermore, the survey incorporates eight municipalities prioritized for their high levels of violence, allowing for a more precise approximation of social dynamics in contexts characterized by extreme exposure to violence against women. It also includes major urban centers, such as the Central District and San Pedro Sula. Nevertheless, due to the specificities of the sampling design, these differences must be interpreted with caution.

Finally, the analysis is predicated on the effective sample of women interviewed in the ENESVMN (*n* = 5,962). Throughout this manuscript, statements in the Abstract, Results, Discussion, and Conclusions refer to “the analyzed ENESVMN sample” rather than to women in Honduras generally. All data presented were processed using IBM^®^ SPSS^®^ Statistics Software version 27. Supplementary material S1 provides complete executable SPSS syntax, execution instructions and identification of the required data file. It reconstructs both index specifications, all item recodings, normalized sampling weights, classification thresholds, item frequencies, subgroup and municipal tables, partner-item sensitivity analyses, correlations, classification agreement and confidence intervals. The procedure was verified from the source variables in the supplied 5,962-case database, without relying on its previously stored analytical variables.

All fourteen item flags were summed with equal item weights. For Tables 1–3, the applicability-subgroup summaries and the comparison with the historical sixteen-item specification, respondent-level sampling weights were applied using FactorMV normalized to the actual sample size (*n* = 5,962). Normalized weighted counts are not observed respondent counts. The partner-item sensitivity percentages, classification agreement and correlations reported below were calculated without sampling weights; Supplementary material S1 also reports that sensitivity comparison using the same normalized weights as the main tables. These descriptive analyses do not incorporate the survey's full complex design for variance estimation.

The normalized respondent weight was calculated as FactorMV × N/SUM(FactorMV), where *N* = 5,962 and the sum is taken over all analyzed respondents. General proportions and Table 1 item percentages use unrounded weighted sums. Subgroup and municipal cross-tabulations use SPSS COUNT ROUND CELL: each weighted cell count is rounded before calculating row and column percentages. Consequently, sums of rounded cells can differ from separately rounded overall totals. The reported 95% confidence intervals are nominal Wilson intervals calculated from the exact weighted proportions and the actual sample *N*. They reproduce the reported limits but do not account for stratification, clustering or variability in sampling weights.

## Results

### General estimation of social cohesion

#### Sensitivity analysis

We assessed sensitivity to the coding of the two partner-referencing participation items (MC1107_5, MC1107_7). In the sensitivity specification, responses 1 (must ask permission) and 3 (does not go alone, goes with him) were scored 1 for both items. Responses 2 (informs him or asks for his opinion) and 4 (does not need to do anything/does not do it), other responses (code 5), not-applicable responses (code 6), and missing responses were scored 0. This also changes the treatment of missing responses to MC1107_5. The resulting score has an observed range of 3–13; using high ≤ 7, moderate = 8 and low ≥9, the unweighted distribution is 18.3% high, 25.4% moderate and 56.3% low cohesion. It agrees with the primary classification for 82.9% of respondents (4,941 of 5,962). The unweighted continuous scores correlate strongly (Pearson *r* = 0.91; Spearman ρ = 0.91). Applying the main-table normalized weights to the alternative classification gives 19.0% high, 25.2% moderate and 55.8% low cohesion, with 83.2% agreement. Low cohesion remains the modal category under both codings and weighting settings. However, this categorical comparison changes both item coding and classification thresholds; the observed reclassification cannot be attributed to coding alone, and the exact category shares remain sensitive to these analytical choices.

The recommendations that follow are advanced as policy propositions informed by the theoretical and empirical literature cited throughout, not as findings established by the present descriptive analysis. The ENESVMN data used here do not test the causal effects of fiscal policy, institutional reform, or any specific intervention on social cohesion. Each proposal below draws on prior research addressing those questions and applies it to the Honduran context this study describes; where a recommendation depends on evidence external to this study, that evidence is cited, and its applicability to Honduras reflects the authors' own judgment rather than a result demonstrated here.

In an initial specification, the index also included two additional items beyond the fourteen retained here: whether the respondent shares a bedroom with a household member, excluding situations of overcrowding that this study did not examine (DV310_1), and the respondent's self-identified ethnic group (RD401). Sharing a bedroom was considered an indicator of cohesion, particularly among couples (spouses or partners), and sharing living space with others more broadly was read the same way, insofar as it favors proximity and daily interaction. Self-identifying as mestizo/ladino was considered an indicator of cohesion insofar as it reflects historical attempts at national integration in Honduras, although these policies rested on ambiguous categories and were accompanied by discriminatory practices that expose the fragility and limits of that integrationist project (Díaz Arias, 2007; Euraque, 2005).

We excluded both items from the definitive, fourteen-item specification because neither maps as directly onto Mora Salas's (2015) own four dimensions as the retained items do; shared living space is not equivalent to the frequency-of-contact and expectation-of-support items through which his framework operationalizes social bonds and trust, and ethnic self-identification is not equivalent to the perceived-discrimination item through which he operationalizes tolerance under shared values. With these two items included, the historical three-band classification applied to the resulting sixteen-item score (range 4–15, high 4–7/moderate 8–11/low 12–15) yields a normalized-weight distribution of 3.4% high, 67.5% moderate, and 29.1% low cohesion (actual *N* = 5,962), a markedly different distribution from the fourteen-item primary specification reported above. Cross-classifying the two categorical variables, normalized-weight agreement between the fourteen-item and sixteen-item specifications is 59.1% (a weighted count of 3,523.017 out of 5,962). The corresponding unweighted number of respondents in the same category is 3,509; all disagreement is confined to the high/moderate and moderate/low boundaries, with no respondent classified as high under one specification and low under the other, or vice versa. This comparison is reported here as a check on the sensitivity of the classification to item inclusion and the accompanying threshold scheme; it does not indicate that the sixteen-item version is an available alternative primary measure. The analytic syntax for both specifications, including the item-level recoding used to construct each score, is provided in Supplementary material S1.

## Municipal-level estimation of social cohesion

### Discussion and conclusion

#### Discussion of general data

The general distribution—with high cohesion (27.1%) and moderate cohesion (26.6%) roughly balanced, and low cohesion as the largest single category (46.3%)—confirms that cohesion does not operate as a dichotomous condition but rather as a continuum, in alignment with the Mora Salas (2015) framework. High levels of cohesion are concentrated among married or cohabiting women, those aged 30 to 64, and those with higher educational attainment. Conversely, low cohesion is descriptively more prevalent among younger women, women with less schooling, and respondents in the other marital-status group. Cohesion is spread fairly evenly between the high and moderate levels, with low cohesion as the single largest category—a pattern compatible with what Martínez Franzoni et al. (2025) describe as “unstable coexistence,” as values skew toward the lower end of the distribution rather than concentrating at the top.

This interpretation aligns with the perspective of Feinberg et al. (2023), who conceptualize cohesion as a dynamic, non-linear process susceptible to strengthening or deterioration based on the conditions of social interaction. In this case, the inclination of values toward lower levels may indicate that cohesion is strained by adverse structural factors, including violence and inequality. However, following Mora Salas (2015), this reading must be nuanced; inequality is not a defining component of social cohesion but rather an external factor whose impact—whether through erosion or strengthening (p. 127)—must be empirically established.

Disaggregation by educational level demonstrates that low cohesion is concentrated among women with less schooling (59.1% of the low-cohesion group has only primary education), whereas high cohesion is comparatively more associated with higher education (20.8% of the high-cohesion group, vs. 7.0% of the low-cohesion group). This result is consistent with Aruqaj (2023), who identifies education as a factor that fosters social trust, openness, and participation—core components of cohesion.

Regarding marital status, the results indicate that high cohesion is more common among married or cohabiting women, who make up 62.0% of the high-cohesion group, while respondents in the other marital-status group make up a larger share of the low-cohesion group (38.8%) than of the high-cohesion group (29.5%). This pattern is consistent with the family operating as a support for primary social ties, particularly in precarious contexts (Sabillón Casco et al., 2018). In alignment with Mosqueda Tapia (2023), family bonds operate as compensatory mechanisms against structural limitations, reinforcing cooperation and mutual support. Because the other marital-status group pools divorced, separated, single and unknown responses, its distribution cannot establish a pattern specific to single women or distinguish the mechanisms underlying these differences.

These figures describe the composition of each cohesion level by marital status. Within-group prevalence, reported in the Results above, shows low cohesion in 51.5% of the other marital-status group, compared with 44.8% of married or cohabiting women and 33.6% of widowed women. High-cohesion prevalence ranges from 23.0% to 29.5% across these groups. These comparisons are descriptive and cannot establish an independent association, particularly because partner-referencing items are included in the index (see Limitations).

The distribution across age groups reinforces this logic. Young women (aged 15 to 29) make up a larger share of the low-cohesion group (35.2%) than of the high-cohesion group (23.2%), while the 30-to-64 age group makes up the largest share of the high-cohesion group (63.0%). This behavior is consistent with evidence linking cohesion to one's position in the life cycle; intermediate age groups tend to possess greater labor, familial, and institutional stability, which facilitates the consolidation of social networks. In contrast, young women face more significant restrictions in terms of access to resources, social participation, and stability (Briceño-León and Camardiel, 2021; Quiroga Juárez and Villafuerte Valdés, 2023).

As above, this age-group pattern describes the composition of each cohesion level rather than the within-group prevalence of cohesion by age. The within-group prevalence figures reported in the Results above show the same direction: low cohesion is most prevalent among women aged 15–29 (55.4%) and least prevalent among those aged 30–64 (42.4%), followed closely by those aged 65 and older (42.6%), and should be read descriptively rather than as an association established by inferential testing.

Consequently, social cohesion does not emerge as a homogeneous attribute of society but rather as an unequal and contested configuration. This reading aligns with the proposal of Martínez Betancourt and Neira Milian (2021), who conceptualize cohesion as a historical and contextual construction shaped by social tensions.

#### Discussion of municipal breakdown

From a territorial perspective, the results must be interpreted with caution in light of the survey's sampling design. While an apparent fragmentation is observed among municipalities—where major urban centers, such as the Central District and San Pedro Sula, concentrate higher proportions of high and moderate cohesion, whereas the aggregate labeled as “remaining municipalities” shows a strong concentration of low cohesion (66.1%)—this difference cannot be read exclusively as a reflection of territorial structural inequalities. Indeed, the survey intentionally prioritized a set of municipalities with high levels of violence, granting them specific representativeness and separating them from the rest of the national territory [National Institute of Statistics (INE), 2022].

Consequently, the “remaining municipalities” group functions primarily as a residual category encompassing high territorial heterogeneity, considering that Honduras consists of 298 municipalities. Thus, its relative weight in the low cohesion category may be influenced both by its aggregate size and the lack of internal disaggregation. Therefore, rather than conclusively evidencing lower cohesion in these territories, the results suggest the need to interpret this category as a broad analytical space where multiple social realities converge without being captured differentially.

Nevertheless, this configuration allows for an analytical advantage regarding the municipal segregation introduced by the survey design. Rather than focusing solely on the magnitude of low cohesion in the “remaining municipalities,” it is more pertinent to compare the prioritized municipalities—characterized by high violence—with the major urban centers and the remaining aggregate. This facilitates the identification of how social cohesion behaves across differentiated contexts of risk and institutional intervention. Within this framework, the findings partially align with Candia Cid et al. (2022) regarding the relevance of the subnational scale; however, they also demonstrate that this scale is mediated by methodological decisions that condition the interpretation of territorial inequalities.

#### Policy implications

To improve social cohesion levels, public management must transcend traditional welfarism and adopt a multidimensional approach that integrates material and subjective dimensions. A primary recommendation is the reduction of socioeconomic inequalities, as empirical evidence demonstrates that income inequity significantly undermines institutional trust and openness toward others (Aruqaj, 2023; Mora Salas, 2015, p. 126).

The comparative studies cited throughout this article differ in measurement, sampling, and design, and these differences condition how directly their findings can be compared with the present estimation. Aruqaj (2023) develops a multi-country index from harmonized national surveys; Candia Cid et al. (2022) validate a psychometric scale through primary data collection at the municipal level in Chile; Briceño-León and Camardiel (2021) rely on a purpose-built victimization and perception survey; and Quiroga Juárez and Villafuerte Valdés (2023) use AmericasBarometer perception data. None of these designs matches the present study's reliance on proxy items drawn from a survey not designed to measure cohesion, so the comparisons offered here are read as broadly consistent directions in the literature rather than as numerically comparable estimates.

In this regard, public policy cannot be limited to resource provision but must instead be oriented toward creating a transparent institutional architecture that generates legitimacy. Evidence suggests that citizen evaluation of the police force and the criminal justice system is the factor with the greatest direct impact on social cohesion, even surpassing the effect of fear of crime (Briceño-León and Camardiel, 2021).

Furthermore, the cited evidence suggests it would be valuable for decision-makers to prioritize the municipal and local scales in their interventions. As the smallest political-administrative unit, the municipality allows for more precise monitoring of social ties and community support expectations, facilitating non-normative measurements that capture the reality of individuals within their immediate environment (Candia Cid et al., 2022). For these local programs to succeed, management must avoid the logic of veiled clientelism, which fractures neighborhood organizations and reproduces distrust by benefiting only specific groups or leaders close to the administration (Mosqueda Tapia, 2023).

Building on this evidence, another policy proposition worth considering is investment in infrastructure for volunteering and citizen participation. Longitudinal studies confirm a bidirectional causal relationship: fostering volunteer activities not only improves community connection (horizontal cohesion) but also increases trust in national institutions and the government (vertical cohesion) (Davies et al., 2024). Nevertheless, this participation should not be passive; the state must act as a facilitating agent that recognizes individuals as active and competent subjects in policy design and evaluation, promoting participatory governance that aligns governmental objectives with the actual demands of society (Martínez Betancourt and Neira Milian, 2021).

Finally, drawing on this literature, we propose that public management integrate dignified work as a catalyst for cohesion. Employment should not be seen merely as a means of income but as a source of self-fulfillment and mutual aid that reduces alienation and strengthens the social fabric through the creation of social value (Martínez Betancourt and Neira Milian, 2021). In high-violence contexts, it is recommended that authorities focus on mitigating everyday or minor crimes (such as robberies and neighborhood assaults), as their daily frequency has a much greater dissolving impact on coexistence than large-scale crimes, being the factors that most significantly fracture the “social cement” of daily life (Briceño-León and Camardiel, 2021).

In extending these recommendations to survivor-centered support, safe institutional access, and economic autonomy, we caution against assuming that stronger family or partner proximity is protective in itself. Where violence occurs within the household, closer proximity can coincide with greater risk rather than greater cohesion.

#### Limitations

This study has six main limitations. First, construct validity. The cohesion index is built from proxy items in a survey not designed to measure cohesion and has not been validated psychometrically; several retained items (social security coverage, police/court help-seeking, street lighting) are better understood as institutional-access proxies than as direct measures of interpersonal cohesion. Second, routing and conditional items. Several index items were asked only of women reporting a prior experience, such as community problems or violence. Missing, routed-out and not-applicable responses were assigned scores using the item-specific rules in Data and Methods, so fourteen computed flags do not imply fourteen observed substantive responses. Among respondents with substantive responses to MC1107_5 (2,511 actual cases), the normalized-weight distribution was 36.4% high, 27.8% moderate and 35.8% low cohesion. Among those with not-applicable or missing responses (3,451 actual cases), it was 20.8%, 25.8%, and 53.4%, respectively. These are applicability subgroups, not fourteen-item and thirteen-item scores. Their differences cannot isolate the contribution of scoring rules from differences in respondent characteristics and should be interpreted descriptively. Third, circularity in subgroup comparisons. Because two retained items reference a spouse or partner, comparisons by marital status risk partly reflecting the index's own construction rather than an independent association.

We have not been able to construct a validated sensitivity index excluding every partner-referencing item, and we therefore restrict our interpretation of the marital-status comparisons in the Section Discussion to a descriptive one rather than an independent finding. Fourth, incomplete adjustment for the complex sampling design. Although normalized sampling weights were applied to the main descriptive estimates, the reported confidence intervals do not account for stratification, clustering, or unequal sampling weights. The partner-item sensitivity results were calculated without sampling weights, with a weighted comparison provided in Supplementary material S1. Fifth, threshold and classification uncertainty. The thresholds separating high, moderate and low cohesion are explicit analytic choices rather than an externally validated typology. The primary, partner-item sensitivity and historical sixteen-item specifications use different cut-points, so their categorical comparisons reflect changes in both the score and the classification scheme.

The partner-item sensitivity comparison gives 82.9% unweighted agreement and Pearson *r* = 0.91 for the continuous scores; its alternative distribution still places the largest share in low cohesion. This does not establish that the precise percentages in Tables 2, 3 are invariant to coding or thresholds. Sixth, reproducibility and data dependence. Supplementary material S1 provides the complete analytical syntax, including item recoding, weighting, classification thresholds, sensitivity analyses and confidence-interval calculations. Reproduction of the reported results requires the corresponding ENESVMN data file and the same coding and rounding settings. Computational reproducibility does not, however, establish the construct validity of the proxy index or the validity of its classification thresholds.

## Conclusions

Within the analyzed ENESVMN sample (*n* = 5,962), the normalized-weight descriptive estimation shows that, on the fourteen-item cohesion index used as this study's primary measure, 27.1% of women score high, 26.6% score moderate, and 46.3% score low, with low cohesion as the single largest category; the composition of cohesion groups differs by education, marital status and age. Married or cohabiting women and those aged 30–64 constitute the largest shares of the high-cohesion group, while young and less educated women account for larger shares of the low-cohesion group than of the high-cohesion group. These statements concern group composition; within-group prevalence is reported separately in the Section Results. These are the findings the descriptive, cross-sectional, proxy-based design of this study can support. The interpretation that follows situates these findings within the theoretical and policy literature; it is presented as literature-grounded argument, not as a further empirical result of this analysis.

At a theoretical level, in contexts such as Honduras, it is not possible to identify an institutionalized form of “expansive cohesion.” This is because the state was configured under an exclusionary logic that historically restricted social protection to elitist arrangements. This trajectory, characteristic of a late and truncated modernization, impeded the consolidation of a balanced link between the labor market, social policy, and wellbeing. Within this framework, public function operates under a “policy of absence,” with a residual state presence in distribution, market regulation, and gender co-responsibility, thereby shifting social risks toward households—and particularly toward women. This is compatible with what the literature describes as “unstable coexistence,” in which cohesion is unevenly distributed across the population and a plurality of women fall into the low-cohesion category; because this is a single cross-sectional wave, the data do not themselves establish a downward trend, and no temporal claim is made here.

From a methodological perspective, the estimation of social cohesion based on the ENESVMN constituted a pragmatic response to the absence of instruments specifically designed to capture this phenomenon. Even so, a functional gradation was achieved in the absence of psychometrically validated scales. While the use of secondary sources implies certain limitations, its strength lies in the fact that the adopted approach approximates social cohesion from the lived experiences of women in contexts of violence, providing a situated reading of the phenomenon. Consequently, it is recommended to move toward the institutionalization of specific, valid, and periodic measurement instruments.

In terms of public policy, we advance a proposition rather than a further empirical finding of this study. Consistent with the welfare-regime literature cited throughout, we argue that low social cohesion in Honduras is unlikely to be reversed without a structural transformation of the state's role, particularly regarding its redistributive capacity. The persistence of a regressive fiscal regime, which maintains broad exemptions for the most favored economic sectors, limits resource mobilization and weakens the possibility of constructing a rights-based welfare system. In this context, social policy ceases to operate as a guarantee mechanism and is reduced to the fragmented management of poverty, reproducing a logic of state charity.

These current fiscal policy decisions not only restrict the provision of essential public goods but also contribute to the erosion of the social contract. Given the state's inability to guarantee security and effective protection, forms of clandestine welfare emerge which, while offering immediate responses, undermine institutional legitimacy and deepen social fragmentation. Therefore, a social cohesion policy requires reconfiguring the link between revenue and rights, moving toward a fiscal system that binds high-income sectors to collective wellbeing. We argue that transitioning from a residual state to one with a real capacity for protection, capable of sustaining universal services in key areas such as health, education, and care, would require this articulation. This proposition, like the recommendations in the Policy Implications section above, is a theoretically informed argument rather than a result tested by the present cross-sectional, proxy-based analysis, and its applicability should be read together with the Limitations discussed above.

## Data Availability

The original contributions presented in the study are included in the article/Supplementary material, further inquiries can be directed to the corresponding author.

## Footnotes

1 The database was publicly available at https://ine.gob.hn/bases-de-datos/. Following the change of government in Honduras in 2026, this download was disabled. Readers wishing to consult the microdata may request the file directly from the authors.
