# Supplementary material for: Social cohesion in contexts of violence against women: evidence from Honduras and implications for public policy
Source: Front Sociol. 2026 Oct 7;11:1925726. doi: 10.3389/fsoc.2026.1925726 (PMC13683096; doi:10.3389/fsoc.2026.1925726)
Supplement: Supplementary file 1 [file Data_Sheet_1.pdf]

Supplementary Material S1

"Social Cohesion in Contexts of Violence Against Women: Evidence from Honduras and Implications for Public Policy"

Software and required data

IBM SPSS Statistics 27. No Python, R or extension commands are required to run the syntax in SPSS. The input is **Encuesta sobre violencia-ENESVM.sav**, the analysis file containing 5,962 cases and 1,506 variables. Only the 21 required source variables are retained on import; all analytical flags, scores, categories and normalized weights are recomputed.

Respondent weights and rounding

Equal item weights are distinct from respondent-level sampling weights. The latter are calculated as FactorMV x N / SUM(FactorMV), where N = 5,962. All source weights are positive and nonmissing; SUM(FactorMV) = 3,521,687.0053539, and the normalized weights sum to 5,962.

Tables 1-3, the applicability-subgroup summaries and the historical sixteen-item comparison use normalized respondent weights. The reported partner-item sensitivity percentages, agreement count and correlations are unweighted. The syntax additionally reports that sensitivity comparison under the main-table weights.

General estimates and Table 1 item percentages use unrounded weighted sums. Subgroup and municipal cross-tabulations use COUNT ROUND CELL: weighted cells are rounded before row and column percentages are calculated. Sums of rounded cells may differ from separately rounded overall totals.
Weighted counts are not observed respondent counts. Exact fractional counts are also provided.

Subgroups and confidence intervals

Education uses 0-6, 7-12 and 13-20 years; other values are excluded from that table. The other marital-status group pools divorced, separated, single and unknown responses (EstCivil codes 3, 4, 6 and 9). Municipalities use the source MUNPRIORIZADOS grouping.

The 95% intervals use the Wilson formula with the exact weighted proportion and the actual sample N. They are nominal descriptive intervals and do not account for stratification, clustering or unequal sampling weights. They should not be interpreted as complex-survey confidence intervals.

Numerical verification checkpoints

The complete procedure was executed in SPSS 27 from the retained source variables. All fourteen primary flags, both primary and historical scores and classifications, the applicability indicator and the normalized weights matched the stored analytical variables case by case. The 160 numerical

entries checked in Tables 1-3, including displayed counts, percentages and interval limits, were reproduced.

Primary category	Actual cases	Normalized weighted count	Percent	Nominal 95% CI
High	1,570	1,617.694611	27.1	26.02-28.28
Moderate	1,627	1,586.091833	26.6	25.50-27.74
Low	2,765	2,758.213555	46.3	45.00-47.53

Sensitivity checkpoint	Verified result
Unweighted partner-item distribution	1,089 high; 1,515 moderate; 3,358 low. Percentages: 18.3%, 25.4%, 56.3%.
Unweighted partner-item agreement	4,941 of 5,962 cases, or 82.9%.
Continuous-score correlations	Pearson r = 0.9121135; Spearman rho = 0.9080557.
Weighted partner-item comparison	19.0% high; 25.2% moderate; 55.8% low. Agreement: 83.2%.
Historical sixteen-item comparison	Weighted distribution: 3.4%, 67.5%, 29.1%. Weighted agreement: 59.1% (3,523.017192 normalized weighted cases).<br>Actual agreement count: 3,509.<br>No high/low extreme disagreement.

Applicability subgroup checks

Substantive responses to MC1107_5: **2,511 actual cases**; weighted sum 2,414.652078; weighted high/moderate/low percentages 36.4%, 27.8%, 35.8%. Missing or not-applicable responses: 3,451 actual cases; weighted sum 3,547.347922; percentages 20.8%, 25.8%, 53.4%. All 5,962 respondents have fourteen computed flags in the primary index.

The separately rounded cells in the applicability cross-tabulation sum to 2,415 and 3,548. Their total of 5,963 is a rounding artifact, not an extra respondent.

Complete SPSS syntax

```
* Encoding: UTF-8.
* SUPPLEMENTARY MATERIAL S1 -- COMPLETE ANALYTICAL SYNTAX.
* Social Cohesion in Contexts of Violence Against Women.
* Verified against the supplied 5962-case SAV in IBM SPSS Statistics 27.
* * BEFORE RUNNING: set the SPSS working directory to a writable folder.
* Place the supplied SAV in that folder, or edit the FILE HANDLE below.
* Example: CD 'C:\replication'.
* Run this entire file, not isolated selections.
* Outputs: S1_results.xml and three aggregate CSV files in that folder.* No
  respondent-level data are exported and the input SAV is never overwritten.
* * ANALYTICAL SETTINGS.
* Main tables, item frequencies, applicability subgroups, and the 16-item*
  comparison use normalized respondent weights.
* The partner-item sensitivity is reported without respondent weights.
* A weighted sensitivity comparison is also provided.
* MC1107_5 codes 2-6 score 0; code 1 and missing responses score 1.
* All 14 computed flags contribute to every primary score.
* The applicability indicator describes response status, not score length.
* * 1. INPUT AND EXPLICIT SESSION STATE.
FILE HANDLE S1DATA /NAME='Encuesta sobre violencia-ENESVM.sav'.
GET FILE=S1DATA
 /KEEP=HL3 ACOM553 ACOM554_1 ACOM549 AE207_1 BAYUD_VIO_PN
 BAYUD_VIO_JZ MC1102 MC1107_5 ACOM555_1 DV306A DISCR_LAB
 VTC1204 MC1107_7 DV310_1 RD401 anosest EstCivil HL6
MUNPRIORIZADOS FactorMV.
DATASET NAME S1Cases.
FILTER OFF.
USE ALL.
SPLIT FILE OFF.
WEIGHT OFF.
SET PRINTBACK=ON MPRINT=ON DECIMAL=DOT.
OMS /SELECT ALL
 /DESTINATION FORMAT=OXML
OUTFILE='S1_results.xml'  /TAG='S1'.

*         Only source variables are retained; no pre-existing
analytical variables* or saved weight variable are used to
construct the results.
COMPUTE S1_one=1.
```

```
AGGREGATE OUTFILE=*
MODE=ADDVARIABLES /BREAK=  /S1_N=N
/S1_sumfactor=SUM(FactorMV).
COMPUTE S1_weight=FactorMV*S1_N/S1_sumfactor.
VARIABLE LABELS S1_weight 'FactorMV normalized to the observed case
count'.
COMPUTE S1_badweight=0.
IF (MISSING(FactorMV) OR FactorMV<=0) S1_badweight=1.
FREQUENCIES VARIABLES=S1_badweight.
DESCRIPTIVES VARIABLES=S1_N S1_sumfactor S1_weight
/STATISTICS=MEAN MIN MAX SUM.
*         Expected: N=5962; sumfactor approximately
3521687.005;* positive nonmissing weights; sum of
S1_weight=5962.

* 2. FOURTEEN PRIMARY FLAGS: 0=COHESION, 1=NO COHESION.
* ELSE includes system-missing responses; explicit codes take precedence.
* These mappings reproduce the saved item flags in the supplied database.
RECODE HL3 (1=0) (ELSE=1) INTO S1_i01.
RECODE ACOM553 (1=0) (ELSE=1) INTO S1_i02.
RECODE ACOM554_1 (1 THRU 3=0) (ELSE=1) INTO S1_i03.
RECODE ACOM549 (1=0) (ELSE=1) INTO S1_i04.
* Social security: code 11 is NONE; code 12 is OTHER COVERAGE.
RECODE AE207_1 (1 THRU 10=0) (12=0) (ELSE=1) INTO S1_i05.
RECODE BAYUD_VIO_PN (1=0) (ELSE=1) INTO S1_i06.
RECODE BAYUD_VIO_JZ (1=0) (ELSE=1) INTO S1_i07.
RECODE MC1102 (1=0) (ELSE=1) INTO S1_i08.
* Primary coding of participation, including missing responses.
RECODE MC1107_5 (2 THRU 6=0) (ELSE=1) INTO S1_i09.
RECODE ACOM555_1 (1 THRU 8=1) (ELSE=0) INTO S1_i10.
RECODE DV306A (1=0) (ELSE=1) INTO S1_i11.
RECODE DISCR_LAB (0=0) (ELSE=1) INTO S1_i12.
RECODE VTC1204 (7=0) (ELSE=1) INTO S1_i13.
RECODE MC1107_7 (1,2,5=1) (ELSE=0) INTO S1_i14.
VARIABLE LABELS
 S1_i01 'HL3: household head'
 S1_i02 'ACOM553: talks or seeks help with community problems'
 S1_i03 'ACOM554_1: relatives, friends or neighbours'
 S1_i04 'ACOM549: received help'
 S1_i05 'AE207_1: social security or other listed coverage'
 S1_i06 'BAYUD_VIO_PN: sought police help'
 S1_i07 'BAYUD_VIO_JZ: sought court help'
```

```
 S1_i08 'MC1102: knows laws protecting women'
 S1_i09 'MC1107_5: primary participation coding'
 S1_i10 'ACOM555_1: consequences of experienced situations'
 S1_i11 'DV306A: street lighting'
 S1_i12 'DISCR_LAB: labour discrimination'
 S1_i13 'VTC1204: equitable domestic workload'
S1_i14 'MC1107_7: primary voting coding'.
VALUE LABELS S1_i01 TO S1_i14 0 'Cohesion' 1 'No cohesion'.
COMPUTE S1_flags_present=NVALID(S1_i01 TO S1_i14).
COMPUTE S1_score14=SUM.14(S1_i01 TO S1_i14).
RECODE S1_score14 (4 THRU 8=1) (9=2) (10 THRU 14=3)
(ELSE=SYSMIS) INTO S1_level14.
VARIABLE LABELS S1_score14 'Primary 14-item no-cohesion score'.
* Fixed analysis cut-points; observed range is verified, not assumed.
* No automatic threshold selection is performed.

* MC1107_5 applicability subgroup only; does not modify any score.
COMPUTE S1_applicable=0.
IF (ANY(MC1107_5,1,2,3,4,5)) S1_applicable=1.
VALUE LABELS S1_applicable
 0 'MC1107_5 missing or not
applicable'  1 'MC1107_5 substantive
response 1-5'.

* 3. SIXTEEN-ITEM HISTORICAL SPECIFICATION.
RECODE DV310_1 (1=0) (ELSE=1) INTO S1_bedroom.
RECODE RD401 (10=0) (ELSE=1) INTO S1_ethnicity.
COMPUTE S1_score16=S1_score14+S1_bedroom+S1_ethnicity.
RECODE S1_score16 (4 THRU 7=1) (8 THRU 11=2) (12 THRU 16=3)
(ELSE=SYSMIS) INTO S1_level16.
COMPUTE S1_agree16=(S1_level14=S1_level16).
COMPUTE S1_extreme16=(ABS(S1_level14-S1_level16)=2).

* 4. PARTNER-ITEM SENSITIVITY ANALYSIS.
* Restrictive responses 1 and 3 score 1; responses 2 and 4 score 0.
* Other (5), not applicable (6), and missing responses score 0.
* This changes both the coding and the classification thresholds.
RECODE MC1107_5 MC1107_7 (1,3=1)
(ELSE=0)  INTO S1_part_sens
S1_vote_sens.
COMPUTE S1_score_sens=S1_score14-S1_i09-S1_i14
+S1_part_sens+S1_vote_sens.
```

```
RECODE S1_score_sens (0 THRU 7=1) (8=2) (9 THRU 14=3)
(ELSE=SYSMIS) INTO S1_level_sens.
COMPUTE S1_agree_sens=(S1_level14=S1_level_sens).
VALUE LABELS S1_level14 S1_level16
S1_level_sens  1 'High' 2 'Moderate' 3 'Low'.
VALUE LABELS S1_agree16 S1_agree_sens 0 'Different' 1 'Same'.

* 5. SUBGROUPS USED IN TABLES 2 AND 3.
RECODE anosest (0 THRU 6=1) (7 THRU 12=2) (13 THRU 20=3)
(ELSE=SYSMIS) INTO S1_education.
VALUE LABELS S1_education 1 'Primary' 2 'Secondary' 3 'Higher'.
* Other marital status pools divorced, separated, single and unknown* responses
  (codes 3,4,6,9); all component categories are labelled below.
RECODE EstCivil (1,2=1) (3,4,6,9=2) (5=3)
(ELSE=SYSMIS)  INTO S1_marital.
VALUE LABELS S1_marital 1
'Married/cohabiting'  2
'Divorced/separated/single/unknown' 3 'Widowed'.
RECODE HL6 (15 THRU 29=1) (30 THRU 64=2) (65 THRU
HI=3)  (ELSE=SYSMIS) INTO S1_age.
VALUE LABELS S1_age 1 '15-29' 2 '30-64' 3 '65 and older'.
* MUNPRIORIZADOS is the source grouping: 0=remaining, 1-10=named cities.
* Retain its labels and codes from the supplied SAV.
EXECUTE.

* 6. UNWEIGHTED CHECKS AND PARTNER-ITEM SENSITIVITY.
WEIGHT OFF.
TITLE 'S1 A: Unweighted data checks and partner-item sensitivity'.
FREQUENCIES VARIABLES=S1_flags_present S1_applicable
 S1_score14 S1_score16 S1_score_sens S1_level14 S1_level_sens
S1_agree_sens S1_extreme16.
CROSSTABS /TABLES=S1_level14 BY S1_level_sens
/CELLS=COUNT ROW COLUMN TOTAL /COUNT
ASIS.
CORRELATIONS /VARIABLES=S1_score14 S1_score_sens
/PRINT=TWOTAIL /MISSING=PAIRWISE.
NONPAR CORR /VARIABLES=S1_score14 S1_score_sens
/PRINT=SPEARMAN TWOTAIL /MISSING=PAIRWISE.
* Correlations reproduce descriptive r and rho; p values are not manuscript
* inferential findings. Expected sensitivity counts: 1089,1515,3358.

* Expected same-category count=4941; Pearson=.912114; Spearman=.908056.
```

```
* 7. MANUSCRIPT MAIN RESULTS WITH NORMALIZED SAMPLING
  WEIGHTS.
WEIGHT BY S1_weight.
TITLE 'S1 B: Weighted item frequencies and Tables 2 and 3'.
FREQUENCIES VARIABLES=S1_i01 TO S1_i14 S1_level14 S1_level16.
* COLUMN = composition of each cohesion level; ROW = within-group
  prevalence.
* ROUND CELL reproduces the rounded cell counts in the supplied tables.
CROSSTABS /TABLES=S1_education S1_marital S1_age BY
S1_level14  /CELLS=COUNT ROW COLUMN /COUNT ROUND
CELL.
CROSSTABS /TABLES=MUNPRIORIZADOS BY
S1_level14  /CELLS=COUNT ROW COLUMN
/COUNT ROUND CELL.
* Additional exact fractional counts make normalization and rounding visible.
CROSSTABS /TABLES=S1_education S1_marital S1_age BY
S1_level14  /CELLS=COUNT ROW COLUMN /COUNT ASIS.
CROSSTABS /TABLES=MUNPRIORIZADOS BY
S1_level14  /CELLS=COUNT ROW COLUMN
/COUNT ASIS.

TITLE 'S1 C: Historical index and applicability subgroups'.
CROSSTABS /TABLES=S1_level14 BY S1_level16
/CELLS=COUNT ROW COLUMN TOTAL
/COUNT ASIS.
FREQUENCIES VARIABLES=S1_agree16 S1_applicable.
CROSSTABS /TABLES=S1_applicable BY S1_level14
/CELLS=COUNT ROW COLUMN /COUNT ASIS.
CROSSTABS /TABLES=S1_applicable BY S1_level14
/CELLS=COUNT ROW COLUMN /COUNT ROUND
CELL.
* Historical agreement: weighted sum=3523.017 (59.0912 percent);* the actual
  unweighted same-category count is 3509, not 3523 respondents.
* Applicability: weighted sums=2414.652 and 3547.348 (40.5 and 59.5 percent);*
  actual case counts are 2511 and 3451, not counts of 14-/13-item scores.
* Summing ROUND CELL counts yields subgroup totals 2415 and 3548;* their
  total of 5963 is a cell-rounding artifact, not an extra respondent.

TITLE 'S1 D: Sensitivity under main-table weights'.
FREQUENCIES VARIABLES=S1_level14 S1_level_sens S1_agree_sens.
CROSSTABS /TABLES=S1_level14 BY S1_level_sens
/CELLS=COUNT ROW COLUMN TOTAL /COUNT
ASIS.
```

```
* Weighted sensitivity: 19.0,25.2,55.8 percent; agreement=83.1690 percent.
* This provides sensitivity results under the main-table weights.
WEIGHT OFF.

* 8. GENERAL ESTIMATES AND NOMINAL WILSON INTERVALS.
* Use unrounded weighted counts for proportions, actual N=5962 for Wilson.
* These calculations reproduce the printed 95 percent intervals to 2 decimals.
* They do NOT account for stratification, clusters or weight variability.
* Treat as nominal descriptive intervals, not complex-survey confidence limits.
DATASET COPY S1General.
DATASET ACTIVATE S1General.
AGGREGATE OUTFILE=* /BREAK=S1_level14
/actual_n=N /weighted_n=SUM(S1_weight)
/sample_n=MAX(S1_N).
COMPUTE proportion=weighted_n/sample_n.
COMPUTE percent=100*proportion.
COMPUTE displayed_n=RND(weighted_n).
COMPUTE z=IDF.NORMAL(.975,0,1).
COMPUTE denominator=1+z**2/sample_n.

COMPUTE centre=(proportion+z**2/(2*sample_n))/denominator.
COMPUTE halfwidth=z*SQRT(proportion*(1-proportion)/sample_n
+z**2/(4*sample_n**2))/denominator.
COMPUTE lower95=100*(centre-halfwidth).
COMPUTE upper95=100*(centre+halfwidth).
FORMATS actual_n displayed_n sample_n (F8.0)
weighted_n (F14.6) percent lower95 upper95 (F9.2). TITLE
'S1 E: General estimates and nominal Wilson intervals'.
LIST VARIABLES=S1_level14 actual_n weighted_n displayed_n percent lower95
upper95.
SAVE TRANSLATE OUTFILE='S1_general_estimates.csv'
 /TYPE=CSV /REPLACE /FIELDNAMES /CELLS=VALUES
/KEEP=S1_level14 actual_n weighted_n displayed_n sample_n percent lower95
upper95.
DATASET ACTIVATE S1Cases.
DATASET CLOSE S1General.

* 9. AGGREGATE EXPORTS FOR INDEPENDENT CHECKING.
DATASET COPY S1Cross.
DATASET ACTIVATE S1Cross.
AGGREGATE OUTFILE=* /BREAK=S1_level14 S1_level_sens
S1_level16  /actual_n=N /weighted_n=SUM(S1_weight).
```

```
FORMATS weighted_n (F16.8).
SAVE TRANSLATE
OUTFILE='S1_classification_crosswalk.csv'  /TYPE=CSV
/REPLACE /FIELDNAMES /CELLS=VALUES.
DATASET ACTIVATE S1Cases.
DATASET CLOSE S1Cross.
DATASET COPY S1Groups.
DATASET ACTIVATE S1Groups.
VARSTOCASES /MAKE group_code FROM S1_education S1_marital S1_age
 MUNPRIORIZADOS S1_applicable
/INDEX=group_type /KEEP=S1_level14 S1_weight
/NULL=KEEP.
VALUE LABELS group_type 1 'Education' 2 'Marital status' 3
'Age'  4 'Municipality' 5 'MC1107_5 applicability'.
AGGREGATE OUTFILE=* /BREAK=group_type group_code
S1_level14  /actual_n=N /weighted_n=SUM(S1_weight).
FORMATS weighted_n (F16.8).
SAVE TRANSLATE
OUTFILE='S1_subgroup_counts.csv'  /TYPE=CSV
/REPLACE /FIELDNAMES /CELLS=VALUES.
DATASET ACTIVATE S1Cases.
DATASET CLOSE S1Groups.
WEIGHT OFF.
OMSEND TAG='S1'.
* END. The source file has not been altered or saved over.
```
